# Sustainable High Corrosion Resistance in High-Concentration NaCl Solutions for Refractory High-Entropy Alloys with High Strength and Good Plasticity

**DOI:** 10.3390/e28010105

**Published:** 2026-01-15

**Authors:** Shunhua Chen, Xinxin Liu, Chong Li, Wuji Wang, Xiaokang Yue

**Affiliations:** 1 School of Mechanical Engineering, Hefei University of Technology, Hefei 230009, China; 2 Key Laboratory of Advanced Functional Materials and Devices of Anhui Province, Hefei University of Technology, Hefei 230009, China

**Keywords:** corrosion resistance, refractory high-entropy alloy, single phase, NaCl solution, high strength and plasticity

## Abstract

Among corrosive environments, Cl^−^ is one of the most aggressive anions which can cause electrochemical corrosion and the resultant failures of alloys, and the increase in Cl^−^ concentration will further deteriorate the passive film in many conventional alloys. Here, we report single-phase Nb_25_Mo_25_Ta_25_Ti_20_W_5_C*_x_* (*x* = 0.1, 0.3, 0.8 at.%) refractory high-entropy alloys (RHEAs) with excellent corrosion resistance in high-concentration NaCl solutions. According to potentiodynamic polarization, electrochemical impedance spectroscopy, corroded morphology and the current–time results, the RHEAs demonstrate even better corrosion resistance with the increase in NaCl concentration to 23.5 wt.%, significantly superior to 304 L stainless steel. Typically, the corrosion current density (*i*_corr_) and over-passivation potential (*E*_t_) reached the lowest and highest value, respectively, in the 23.5 wt.% NaCl solution, and the *i*_corr_ (2.36 × 10^−7^ A/cm^2^) of Nb_25_Mo_25_Ta_25_Ti_20_W_5_C_0.1_ alloy is nearly two orders lower than that of 304 L stainless steel (1.75 × 10^−5^ A/cm^2^). The excellent corrosion resistance results from the formation of passive films with fewer defects and more stable oxides. Moreover, with the addition of the appropriate C element, the RHEAs also demonstrated improved strength and plasticity simultaneously, for example, the Nb_25_Mo_25_Ta_25_Ti_20_W_5_C_0.3_ alloy exhibited an average yield strength of 1368 MPa and a plastic strain of 19.7%. The present findings provide useful guidance to address the conflict between the excellent corrosion resistance and high strength of advanced alloys.

## 1. Introduction

Corrosion has always been the key issue of alloys for decades, causing trillions of economic losses globally in each year [1]. Efforts have been devoted to the development of advanced alloys or compositional/structural modifications to achieve desirable corrosion resistance [2,3,4,5]. Among the corrosive environments, Cl^−^ is considered as one of the most aggressive anions which can attack the passive film and result in significant pitting corrosion, leading to the failures of alloys [6,7]. Additionally, the increase in Cl^−^ concentrations will further deteriorate the passive film, causing worse corrosion in varying conventional alloys including steels, aluminum alloys, titanium alloys, and magnesium alloys [8,9,10,11,12,13]. On the other hand, studies have reported the conflict between the corrosion resistance and high strength of alloys, increasing the challenges to develop corrosion-resistant alloys that are strong [3,4,14]. Addressing the corrosion issue for high-strength alloys, especially to prevent the electrochemical corrosion of Cl^−^ in seawater or Cl^−^ containing environments, is therefore crucial for the widespread applications of alloys in marine engineering, power plants, and pipeline engineering.

Recently, refractory high-entropy alloys (RHEAs) allowing multiple refractory elements as principal constituents, demonstrate high strength especially for harsh environments, and thus attract wide research attention [15,16,17]. In addition, some RHEA systems also show good corrosion resistance superior to conventional alloys [18,19]. For example, the Hf_0.5_Nb_0.5_Ta_0.5_Ti_1.5_Zr RHEA was reported to exhibit about one-fifth of the corrosion current density of 316 L stainless steel in the 3.5 wt.% NaCl solution and display an extremely high pitting potential of 8.36 V [18]. TiCrVNb_0.5_Al_0.5_ RHEA displayed a lower corrosion current density (8.91 × 10^−8^ A/cm^2^) and higher breakdown potential (1.91 V) in the 3.5% NaCl solution [19]. The protective oxide layer was easily formed due to the larger amount of corrosion resistance in RHEA, which protected it from electrochemical corrosion. Therefore, the development of RHEAs, which may combine high strength levels with good corrosion resistance, thus provides a possible solution to resolve the Cl^−^ electrochemical corrosion issue for high-strength alloys.

Here, we report Nb_25_Mo_25_Ta_25_Ti_20_W_5_C*_x_* (*x* = 0.1, 0.3, 0.8 at.%) RHEAs with excellent corrosion resistance in NaCl solutions, combined with high strength and good compressive plasticity. More importantly, differing from the deterioration effect in high Cl^−^ concentration electrolytes for conventional alloys, the present RHEAs show improved corrosion resistance with the increase in NaCl concentrations even reaching to 23.5 wt.% (for example, yield strength (*σ*_y_) of 1368 MPa, plastic strain (*ε*_p_) of 19.7%, and pitting potential (*E*_pit_) of 2.6 V in 23.5 wt.% NaCl solution for the Nb_25_Mo_25_Ta_25_Ti_20_W_5_C_0.3_ alloy). The improved corrosion resistance was ascribed to the fewer defects and the formation of stable oxides in the passive film, expanding the voltage range before the occurrence of pitting. The present findings provide useful guidance to address the Cl^−^ electrochemical corrosion issue for the passive films of advanced alloys, and expand their application potential for harsh corrosive environments.

## 2. Materials and Methods

### 2.1. Phase Formation of the RHEAs Based on Empirical Criteria

The criterion based on the empirical parameters of mixing entropy (Δ*S*_mix_), mixing enthalpy (Δ*H*_mix_), and atomic radius difference (*δ*) indicates that the solid solution tends to form in HEAs when 12 J/(mol⋅K) ≤ Δ*S*_mix_ ≤ 17.5J/(mol⋅K), −15 kJ/mol ≤ Δ*H*_mix_ ≤ 5 kJ/mol, and *δ* ≤ 6.5% [20]. The corresponding parameters are calculated by following equations:

(1) $$\Delta S_{mix}=-R\sum_{i=1}^{n}c_{i}lnc_{i}$$

(2) $$\Delta H_{mix}=\sum_{i=1,i\neq j}^{n}4c_{i}c_{j}\Delta H_{ij}$$

(3) $$\delta =\sqrt{\sum_{i=1}^{n}c_{i}{\left(1-\frac{r_{i}}{\bar{r}}\right)}^{2}}$$

where R is the gas constant with the value of 8.3144 J/(mol⋅K), *c*_i_ (*c*_j_) is the atomic percentages of the *i*th (*j*th) element, Δ*H*_ij_ is the mixing enthalpy between the *i*th and *j*th elements, *r*_i_ is the atomic radius of the *i*th element, and $\bar{r}$ is the average atomic radius. Yang et al. [21] defined a new parameter of *Ω*, which considered the combined effect of temperature, Δ*S*_mix_, and Δ*H*_mix_, which can be expressed by the following equation:

(4) $$\Omega =\frac{T_{m}\Delta S_{mix}}{\left|\Delta H_{mix}\right|}$$

It indicates that the solid solution phases favor to form when *Ω* ≥ 1.1 and *δ* ≤ 6.6%. Guo et al. [22] proposed a method to predict the structure of solid solution phases from the valence electron concentration (*VEC*) of HEAs, as follows:

(5) $$VEC=\sum_{i=1}^{n}c_{i}{\left(VEC\right)}_{i}$$

where *VEC*_i_ represents the valence electron concentration of the *i*th element. When *VEC* ≤ 6.87, body-centered cubic-type (BCC-type) phases tend to form; when 6.87 ≤ *VEC* ≤ 8, the BCC-type and face-centered cubic-type (FCC-type) phases are more likely to coexist in the HEAs; when *VEC* ≥ 8, the FCC-type phases favor to form in HEAs. In this work, the empirical parameters of the Nb_25_Mo_25_Ta_25_Ti_20_W_5_C*_x_* RHEAs were calculated, and listed in Table S1. When the x was 0, 0.1, 0.3 and 0.8 at.% (noted as C0, C1, C3, and C8), the calculated Δ*S*_mix_ and *VEC* values have limited variations, implying the formation of same single BCC phase similar to the C0 alloy.

### 2.2. Materials’ Preparation and Characterization

The ingots of the Nb_25_Mo_25_Ta_25_Ti_20_W_5_C*x* RHEAs were fabricated by vacuum arc melting in a water-cooled copper crucible under an argon atmosphere. The raw metals have purities larger than 99.9%, and the C element came from WC powder. The button-like ingots were flipped over and re-melted six times to ensure compositional homogeneity. The phase structure of the RHEAs was examined by the X-ray diffractometer (XRD, PANalytical X-Pert PRO MPD, PANalytical, Almelo, The Netherlands) with Cu-Kα radiation. The microstructure and elemental distribution of the RHEAs were inspected on a Gemini 500 field emission scanning electron microscope (Zeiss, Oberkochen, German), using samples cut from the middle part of the ingots by wire electrical discharge machining (WEDM). The transmission electron microscope (TEM) analysis was conducted on a high-resolution transmission electron microscope (Talos F200X G2, Thermo Fisher Scientific, Waltham, MA, USA). The samples for the microstructure investigation were, firstly, polished using the SiC paper with a grit up to 2000, and then further polished via the colloidal silica suspension. The compressive mechanical properties were examined on a universal mechanical testing machine (C51.105GL, Nss Laboratory Equipment Co., Ltd., Kobe, Japan) under a strain rate of 1 × 10^−4^ s^−1^, and three samples (3 mm × 3 mm × 6 mm) were tested for each condition. The Vickers hardness of the RHEAs was tested on a hardness tester (HV-1000SA, Veiyee, Laizhou, China) at the tested condition of 1000 kgf load with 10 s pressure holding time, where ten points were taken randomly, and the average value was used as the final results. Differential scanning calorimetry (DSC) analysis was conducted on an STA 449 F3 (Netzsch, Selb, Germany) under an argon atmosphere with a heating rate of 10 °C/min, and the testing temperature ranged from 25 °C to 1400 °C.

### 2.3. Electrochemical Tests

Corrosion resistance tests were conducted on an electrochemical workstation (CorrTest, Wuhan, China) in NaCl solutions. To avoid the effect of heterogeneous microstructure on the corrosion behavior, the samples with dimensions of 3 mm × 3 mm × 6 mm were cut from the middle part of the button-like RHEA ingots, where a uniform microstructure existed [23]. A notch was made for inserting the pure copper wire on the 3 mm × 3 mm surface, enabling conductivity, and the other 3 mm × 3 mm surface was used for electrochemical tests at 35 °C. The tested sample surfaces were polished using the SiC paper with a grit up to 2000. The polished samples were set into the epoxy resin for subsequent tests. Before the analyses of the dynamic potential polarization curve and the electrochemical impedance spectrum, the samples were immersed in corresponding NaCl solutions for three hours to ensure the formation of a stable passive film on the sample Zsurface. The electrochemical impedance spectroscopy (EIS) was performed at an open circuit potential with an alternating current (AC) signal amplitude of 10 mV, and the frequency was within the range of 100,000 Hz to 0.01 Hz. The scan rate was 5 mV/s. The corroded sample morphology after electrochemical tests was detected by an SU8020 scanning electron microscope (Hitachi, Tokyo, Japan). The elemental valence states and their concentrations in the passive film were detected by an X-ray photoelectron spectrometer (XPS, Thermo, Waltham, MA, USA). A standard C 1s peak at 284.8 eV was used to calibrate the XPS data. The specimens were immersed for 2.5 h in the 3.5 wt.%, 13.5 wt.%, and 23.5 wt.% NaCl solutions before the XPS analysis.

## 3. Results and Discussion

### 3.1. Phase and Microstructure

**Figure 1:**
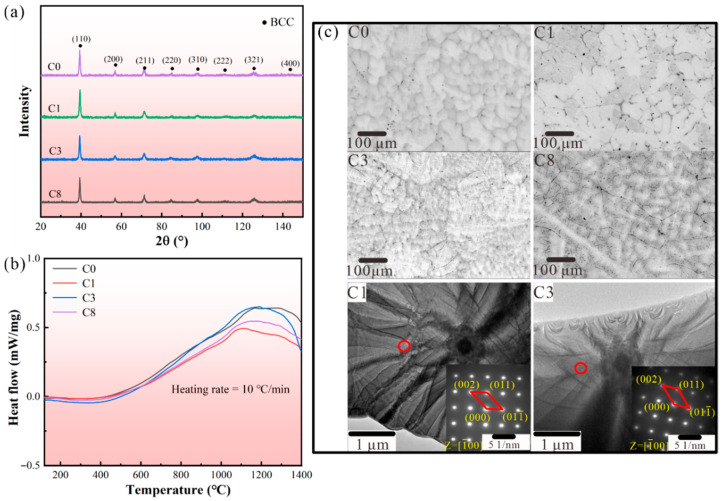
Phase and microstructure characteristics: (**a**) XRD patterns indicating the formation of single BCC phase in C0, C1, C3, and C8 RHEAs. (**b**) DSC curves of the developed RHEAs. (**c**) SEM images showing the microstructure of developed RHEAs, as well as the SAED results for the C1 and C3 alloys, respectively.

A single solid solution phase could contribute to better corrosion resistance for RHEAs [19]. In this work, all Nb_25_Mo_25_Ta_25_Ti_20_W_5_C*x* (*x* = 0, 0.1, 0.3, and 0.8 at.%, noted as C0, C1, C3, and C8, respectively) RHEAs have single BCC phase, as shown in Figure 1a. Based on the solid solution formation criteria of HEAs [20,21], consistent with the XRD results, the developed RHEAs with different C concentrations were all in the range where the solid solution tended to form. The details of the empirical criteria are shown in Methods, and the calculated results are listed in Table S1. As compared with the C0 RHEA, the additional of the non-metal C element did not affect the formation of single BCC phase. In fact, the addition of C can increase the *S*_mix_ [24] and reduce the *VEC* [22] of the RHEA system (Table S1), which benefits the formation of a single BCC phase. The thermal stability of the single phase in these RHEAs was also examined using DSC analysis, and the results are shown in Figure 1b. It can be seen that there was no obvious endothermic/exothermic peak found in the curves ranging from room temperature to 1400 °C, indicating the good phase stability of the developed RHEAs within a large temperature range.

The typical microstructure of the Nb_25_Mo_25_Ta_25_Ti_20_W_5_C*x* RHEAs is given in the SEM images in Figure 1c, showing dendritic structures. Due to the solidification sequence of constituent elements induced by the melting point discrepancy, elemental segregation is generally observed in as-cast RHEAs [25]. With the addition of the C element, obvious boundaries were observed at the interdendritic regions of the C1 alloy (Figure 1c). The energy-dispersive spectroscopy (EDS) results of the C1 alloy indicated that the formation of C-rich black particles concentrated at the obvious boundaries (Figure S1a). The analysis of atomic combinations, determined by the mixing enthalpy (*H*_mix_), reveals that Ti exhibits a relatively lower *H*_mix_ with C in comparison to other constituent elements (*H*_Ti,C_ = −109 kJ/mol, *H*_Nb,C_ = −102 kJ/mol, *H*_Ta,C_ = −101 kJ/mol, *H*_Mo,C_ = −67 kJ/mol, and *H*_W,C_ = −60 kJ/mol), which suggests that the C element is inclined to accumulate at the Ti-rich interdendritic regions [26]. Moreover, due to the sluggish diffusion effects, once the C atom was trapped by Ti atom, it was hard for the C atom to escape from the site due to the various band configurations and lattice potential energy, which resulted in the concentration of C in the interdendrite regions [27]. With further increase in the C content, most doped C seemed to dissolve into the matrix in C3, which possibly promoted the nucleation rate and refined the dendrites. The EDS results presented in Figure S1b indicate that there was a concentration of C at the boundaries. A similar phenomenon was also found in the WNbMoTa RHEA, where a more negative segregation energy of C was utilized to explain the segregation of C at the grain boundary instead of the inner grain [16]. The increased C content may weaken the impeding effect of the sluggish diffusion effect on the atom migration process, promoting the C to dissolve into the boundary. An additional 0.8 at.% doped C led to the formation of larger-sized carbon-rich precipitates within the matrix of C8 (Figure S1c), which suggested that the solubility limit of the alloy matrix for C has reached its limit. Here, with the further increase in the C content, the dissolution of the C element in both the dendritic and interdendritic regions may result in solid solution strengthening and dendrite structure refinement, especially for the C3 alloy (Figure 1c). The bright field TEM image and select area electron diffraction (SAED) patterns of the C1 and C3 RHEAs are also shown in Figure 1c. Both the C1 and C3 RHEAs displayed single BCC phases, which further prove the formation of a single-phase structure even with the C addition. Moreover, the lattice parameters for the C1 and C3 RHEAs, calculated based on the SAED patterns, were 3.235 Å and 3.282 Å, respectively, which further confirmed the enhancement of lattice distortion effect with the addition of more C content.

### 3.2. Corrosion Behavior in NaCl Solutions

**Figure 2:**
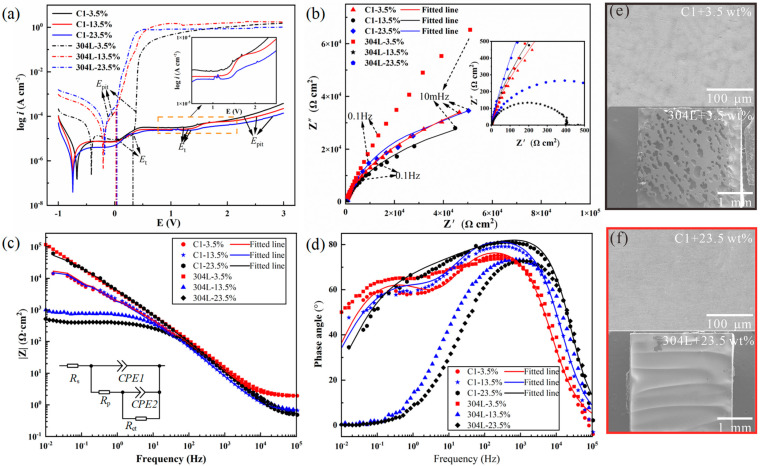
Corrosion testing results: (**a**) Potentiodynamic polarization curves of the C1 alloy and 304 L stainless steel in 3.5 wt.%, 13.5 wt.%, and 23.5 wt.% NaCl solutions at 35 °C. (**b**) Corresponding Nyquist diagrams. (**c**) Bode magnitude plots with inset showing the corresponding EEC model. (**d**) Bode phase plots. (**e**,**f**) Corroded morphology of C1 and 304 L stainless steel in 3.5 wt.% and 23.5 wt.% NaCl solutions, respectively.

The potentiodynamic polarization curves of the typical C1 RHEA and 304 L stainless steel, tested in 3.5 wt.%, 13.5 wt.%, and 23.5 wt.% NaCl solutions, are shown in Figure 2a. The corrosion current density (*i*_corr_), corrosion potential (*E*_corr_), and over-passivation potential (*E*_t_) are summarized in Table S2. In the 23.5 wt.% NaCl solution, the *i*_corr_ (2.36 × 10^−7^ A/cm^2^) of the C1 RHEA is nearly two orders lower than that of 304 L stainless steel (1.75 × 10^−7^ A/cm^2^). Compared with the fluctuating change in *E*_corr_ with the increase in electrolyte concentrations for 304 L stainless steel, the less changed *E*_corr_ in the tested concentration of the C1 alloy demonstrated more stable corrosion resistance. Differing from the sharply rising current density after the *E*_t_ for 304 L stainless steel, an obvious second passive region was observed in the C1 alloy, and the change in current density was moderate, as shown in the insert image in Figure 2a. This phenomenon demonstrated that even beyond *E*_t_, severe pitting may not occur in the C1 RHEA, indicating excellent resistance to pitting. For all C1, C3, and C8 RHEAs (Table S2 and Figure S2), although the *i*_corr_ exhibited a fluctuant change with the increase in NaCl concentration, the *i*_corr_ and *E*_t_ reached the lowest and highest value, respectively, for each RHEA in 23.5 wt.% NaCl solution, demonstrating an enhanced corrosion resistance in the high-concentration NaCl solution. It should be mentioned that although the corrosion current density and pitting potential of all C-doped C1, C3, and C8 RHEAs was slightly worse than the Hf_0.5_Nb_0.5_Ta_0.5_Ti_1.5_Zr [18] or TiCrVNb_0.5_Al_0.5_ [19] alloys in 3.5 wt.% NaCl solution; for the first time, they demonstrated excellent and stable corrosion resistance in even high-concentration NaCl solutions (13.5 wt.% and 23.5 wt.%), superior to many traditional alloys.

The corrosion behavior of the developed RHEAs was further analyzed by electrochemical impedance spectroscopy (EIS). As shown in the Nyquist plots (Figure 2b), the capacitive semicircles of the C1 alloy are almost the same as each other, indicating the insensitivity of the corrosion resistance to the concentration of NaCl solutions. Similar results are also found in other C-doped RHEAs (Figure S3b,c). In line with the results of the potentiodynamic polarization curves, the largest diameter of the semicircle of the C1 RHEA in the 23.5 wt.% NaCl solution indicated the best corrosion resistance. On the contrary, the semicircle diameter of 304 L stainless steel was significantly decreased with the increased solution concentration (Figure 2a), indicating the rapid deterioration of corrosion resistance for 304 L stainless steel. The authors would like to point out that the largest diameter was obtained for 304 L in 3.5% NaCl solution, indicating that such alloy has better corrosion resistance than C1 RHEA in the condition for EIS test; however, the overall corrosion resistance of the alloy should also be determined in other conditions, for example, potentiodynamic polarization curves and corrosive experiments. Nevertheless, in this work, with the increase in NaCl solution concentration, the corrosion resistance of 304 L decreased significantly, while the corrosion resistance of the C1 RHEA almost remained constant. This reflected the stable corrosion resistance of the C1 RHEA. Since the focus of this work is to examine the corrosion behavior and mechanisms of developed RHEAs, the underlying reasons for the corrosion behavior of 304 L are not discussed in detail here.

The Bode plots of the C1 RHEA and 340L stainless steel are shown in Figure 2c. The value of |Z| at 0.1 Hz (recorded as |Z|_0.1_) not only reflects the polarization resistance, but also reveals the corrosion resistance of the tested samples [28]. The |Z|_0.1_ of the C1 RHEA increased in the solution with higher NaCl concentrations, especially in the 23.5 wt.% NaCl solution, while 304 L stainless steel had an obvious decrease. This further proves the excellent corrosion resistance of the C1 RHEA in the high-concentration NaCl solutions. Similar phenomena were also found in the C3 and C8 RHEAs (Figure S3e,f). These findings also indicate the better and stable corrosion behavior of the developed RHEAs as compared with the conventional stainless steel.

From the two peaks in the phase plots of the C1 RHEA (Figure 2d), an equivalent electric circuit (EEC) model of *R*_s_(*CPE*_1_(*R*_p_(*CPE*_2_*R*_ct_))) was used to fit the EIS data of the C1 RHEA, where *R*_s_ is the solution resistant, *CPE* is the constant phase element including the passive film capacitance (*CPE*_1_) and the double layer capacitance (*CPE*_2_), *R*_p_ is the passive film resistance, and *R*_ct_ is the charge transfer resistance (Figure 2c inset). The C3 alloy also showed the same EEC model (Figure S3h and Table S3). Generally, the *R*_p_ represents the impedance of the passive film, and the higher the *R*_p_, the more protective effect of the passive film [29]. As a function of the NaCl concentration, the *R*_ct_ value decreased from 7.97 × 10^4^ Ω·cm^2^ to 6.05 × 10^4^ Ω·cm^2^ when the NaCl concentration increased from 3.5 wt.% to 13.5 wt.%, and then increased to the maximum of 8.61 × 10^4^ Ω·cm^2^ in the 23.5 wt.% NaCl solution. This tendency of the *R*_ct_ to change was consistent with the polarization results [30]. The values of *CPE*_1_ and *CPE*_2_ of C1 RHEA in the 23.5 wt.% NaCl solution were 2.56 × 10^−5^ μF/cm^2^ and 4.23 × 10^−5^ μF/cm^2^, respectively, which were smaller than those in 3.5 wt.% NaCl solution (*CPE*_1_ = 3.48 × 10^−5^ μF/cm^2^; *CPE*_2_ = 7.74 × 10^−5^ μF/cm^2^). The value of *Q* is inversely proportional to the thickness of passive film with similar compositions [30,31,32,33]. The value close to one of n1 indicated that the *CPE*_1_ is closer to the ideal capacitance. The relatively fewer defects in the passive film further improve its protective function against chlorine ions. Therefore, the fewer defects in the passive film of the C1 alloy may be the reason for its better corrosion resistance in the high-concentration NaCl solutions, while for the C8 alloy, the EEC model was changed to *R*_s_(*CPE*_1_(*R*_p_(*CPE*_2_(*R*_sp_(*CR*_ct_)))), which a typical inner and outer layer of passive film generated (Figure S3f). The lower n1 value of C8 (Table S3) demonstrated that the protective property of the passive film was weaker than that of the C1 or C3 alloy (Table S3).

The corroded morphologies of the C1 alloy after potentiodynamic polarization test in 3.5 wt.% and 23.5 wt.% NaCl solutions are given in Figure 2e and Figure 2f, respectively. Compared with the serious pitted and entire corroded surface of 304 L stainless steel in 3.5 wt.% and 23.5 wt.% NaCl solutions, the corroded morphology of all the C1, C3, and C8 RHEAs was intact, and no obvious corroded trace was found, directly showing the superior corrosion resistance (the corroded morphology of the C3 and C8 alloys are shown in Figure S4).

**Figure 3:**
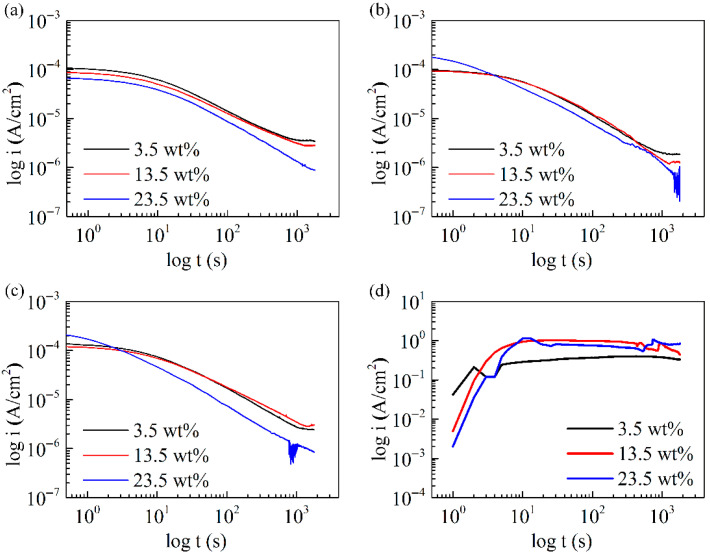
Current–time curves: (**a**–**d**) The change in current density with the increase in measuring time for C1, C3, C8, and 304 L in 3.5 wt.%, 13.5 wt.%, and 23.5 wt.% NaCl solutions, respectively.

According to the passivation range displayed in the potentiodynamic polarization curves (Figure 2a), a certain potential (0.75 V_SCE_) was adopted to generate oxide films for the C1, C3, and C8 RHEAs. In addition, the current–time curves for 304 L stainless steel under the same potential were also carried out. All current–time curves are shown in Figure 3 and Figure S5. For RHEAs, the current density continued to decrease with the increase in measuring time, even after 1800 s. Generally, the formation and dissolution of the oxide film occurred simultaneously, and the decreased current density indicated that the formation rate was higher than the dissolution rate. Therefore, the oxide film could be more impact or thicker during the whole measuring process. In addition, for C1, the current density decreased gradually with the increased electrolyte concentration. For C3 and C8, the current density in 23.5 wt.% NaCl solution was higher first and then lower than that in 3.5 wt.% and 13.5 wt.% NaCl solutions. These findings have further confirmed the sustainable corrosion resistance of the C1, C3, and C8 RHEAs in solutions with various electrolyte concentrations. As shown in Figure S5, although the C0 alloy demonstrated a similar trend to that of the C-doped alloys, it had a lower current density at beginning. Its density in 23.5 wt.% NaCl solution was also lower than that in 3.5 wt.% NaCl solution with increasing measuring time, especially at the time of 1800 s. For 304 L, the current density increased sharply first and then almost kept the same value of 1 A. This signified that the 304 L samples suffered from severe electrochemical corrosion, which showed much worse corrosion resistance than the RHEAs.

### 3.3. Passive Film Characteristics

**Figure 4:**
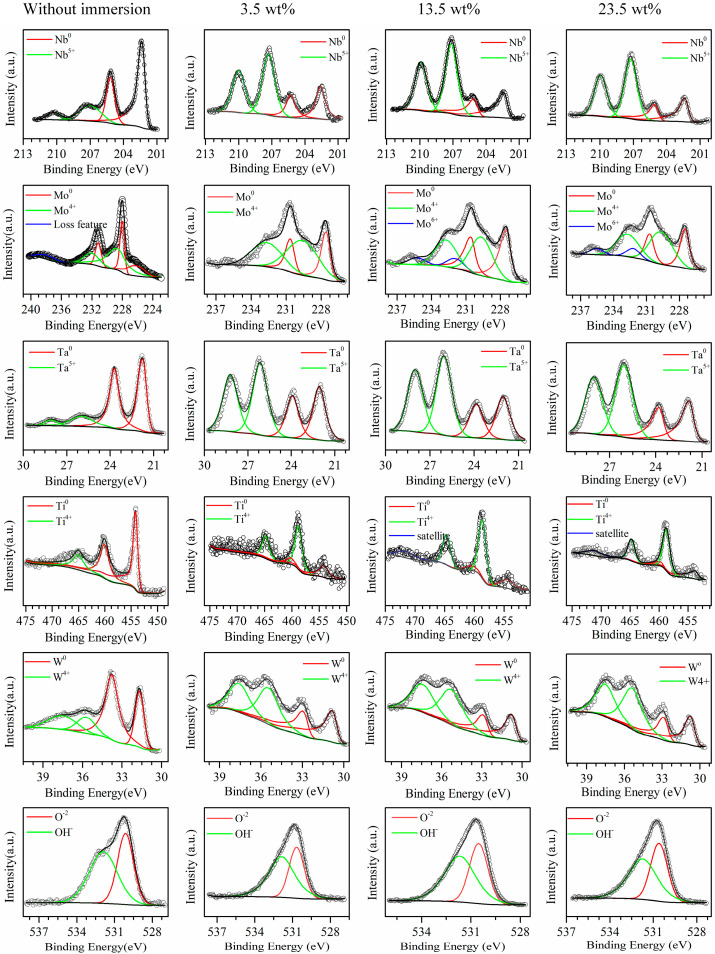
Passive film characteristics: XPS results of the passive film for C1 RHEA without immersing (in air), and the ones immersed in 3.5 wt.%, 13.5 wt.%, and 23.5 wt.% NaCl solutions for 2.5 h.

In order to reveal the underlying reasons for the excellent corrosion resistance of developed RHEAs, X-ray photoelectron spectrometer (XPS) analysis was conducted to investigate the oxidation states of the passive films of the typical C1 alloy. The spectra of the detected elements, such as Mo 3d, Nb 3d, Ta 3d, Ti 2p, and W 4f, are given in Figure 4, and the relative contents of those elements with different valences listed in Table S4. The Nb 3d spectra could be divided into Nb and Nb^5+^_ox_(Nb_2_O_5_) in all solutions. The Nb content decreased little, and the Nb^5+^_ox_(Nb_2_O_5_) content was almost the same with the increase in solution concentration. There were three different valences in the spectra of Mo 3d in all solutions, including Mo, Mo^4+^_ox_ (MoO_2_), and Mo^6+^_ox_ (MoO_3_). It was noticed that the content of the Mo^4+^_ox_ (MoO_2_) in 3.5 wt.% solution was much higher than that in the 13.5 wt.% and 23.5 wt.% solutions, while the Mo^6+^_ox_ only occurred in the latter two solutions. The presence of Mo^6+^_ox_ should play a significant role in the stability improvement of the passive film, enhancing the corrosion resistance [34]. Two peaks were found in the spectra of the Ta element, representing Ta and oxide Ta^5+^ox (Ta_2_O_5_). When the solution concentration increased, the Ta content almost kept the same while the Ta^5+^ox content in 3.5 wt.% solution was lower than that in the 13.5 wt.% and 23.5 wt.% solutions. It was reported that the presence of Ta^5+^_ox_ could contribute to better corrosion resistance [35]. Ti and Ti^4+^_ox_ (TiO_2_) were found in the Ti 2p spectra. Differing from the Ti content, the Ti^4+^_ox_ content in 3.5 wt.% solution was lower than that in 13.5 wt.% and 23.5 wt.% solutions. The presence of the corrosion-resistant element Ti oxide in the passive film is beneficial to the promotion of corrosion resistance [36]. The element W was divided into W and W^4+^_ox_(WO_2_) in all solutions. The content of W was a little higher in the 3.5 wt.% solution than in the other two solutions, while the content of W^4+^_ox_(WO_2_) was almost the same in all solutions. The spectra of O 1s were decomposed into two compositions, such as O^2−^ and OH^−^. Typically, O^2−^ appeared in the form of the oxides of the component elements.

From the XPS analysis, more elements with high valence states are found in the 13.5 wt.% and 23.5 wt.% NaCl solutions. Thus, the formation of more stable oxides in the passive film may be the reason for the enhanced corrosion resistance of C1 in high-NaCl-concentration solutions. On the other hand, the detection of the zero-state metallic elements may be due to the formation of the incomplete passive film. The higher content of zero-state metallic elements in the 3.5 wt.% NaCl solution suggests that the passive film was less compact, decreasing the corrosion resistance. These results are consistent with the calculated n value of the *CPE* of the passive film in the *EEC* model (Table S3). In addition, the XPS of the C1 alloy without NaCl solutions (in air) is also listed in Figure 4, which contained the elements Nb, Mo, Ta, Ti, W, and O. Metal elements with high valence states were also found due to the oxide film formed in air. However, the ratio of metal elements to the oxides with NaCl solutions was obviously larger than that without NaCl solutions. This further proves the formation of the oxide film on the RHEAs in NaCl solutions. As reported in the literature [37,38,39], the change in the content of passive films will affect corrosion resistance. The high-valence oxides, like TiO_2_ or Ta_2_O_5_, have highly charged and stable chemical states, facilitating the formation of a continuous and stable passive film. In addition, the formed passive film with complex composition has a better effect in terms of preventing chloride ion intrusion.

**Figure 5:**
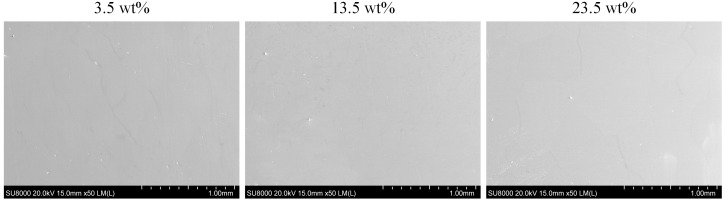
Surface morphology of the C1 RHEA after immersing in NaCl solutions with different concentrations for 2.5 h.

Figure 5 shows the surface of the C1 alloy immersed within NaCl solutions with different concentrations after 2.5 h. For each concentration, no obvious corrosion pit was observed on the whole surface. This result is consistent with the XPS findings that the corrosion resistance of the C1 RHEA in 13.5 wt% or 23.5wt% NaCl solution was as good as that in 3.5 wt%. In addition, the integrity of the surface also confirmed the reliability of the XPS results. These results provide additional evidence for the excellent corrosion resistance of the Nb_25_Mo_25_Ta_25_Ti_20_W_5_C*_x_* RHEAs in high-concentration NaCl solutions. Combined with the corroded morphology shown in Figure 2e,f, the microstructure of these RHEAs showed no obvious changes after corrosion. Further understandings of the effect of microstructural change may also be helpful for elucidating the underlying corrosive mechanisms of these alloys in future. Moreover, for practical applications in harsh corrosive environments, including marine engineering and power plants, other factors such as pH may also affect the corrosion resistance of the RHEAs significantly. This may also be worthy of further investigations in future to better understand comprehensively the corrosion-resistant properties of RHEAs.

### 3.4. Mechanical Properties

**Figure 6:**
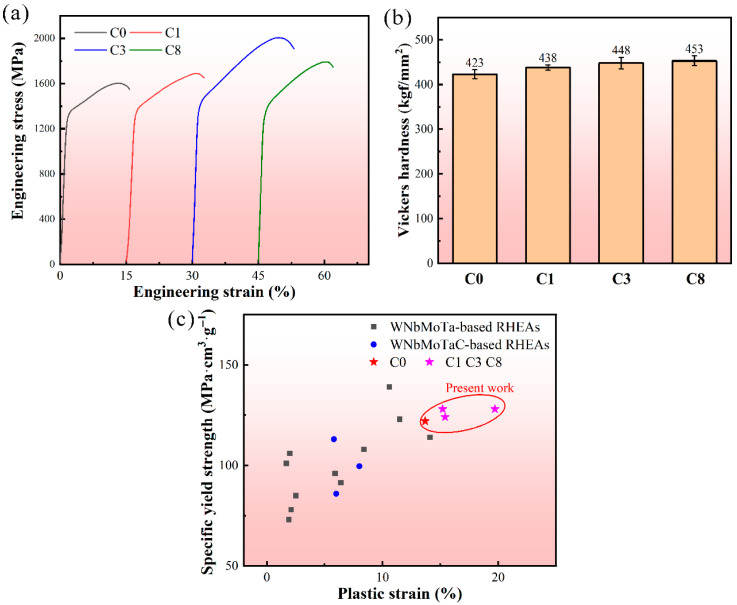
Mechanical properties: (**a**) Compressive engineering stress–strain curves of the C0, C1, C3, and C8 RHEAs. (**b**) Corresponding Vickers hardness data. (**c**) Comparison of the specific yield strength vs. plastic strain plots of single-phase WNbMoTa-based and C-doped WNbMoTa RHEAs, including WNbMoTa [41], WNbMoTaV [41], WNbMoTaTi [42], WNbMoTaVTi [42], WNbMoTaTi*_x_* (*x* = 0, 0.25, 0.5, 0.75, 1) [25], WNbMoTaZr_0.1_ [43], WNbMoTaVZr_0.1_ [44], and (NbMoTaW)_100−*x*_C*_x_*(*x* = 0.05, 0.15, 0.5 at.%) [16], as well as the present C0, C1, C3, and C8 RHEAs.

The engineering compressive strain–stress curves, as well as the Vickers hardness of RHEAs, are shown in Figure 6a,b, and the detailed data of the yield strength, maximum strength, and fracture plasticity are shown in Table S5. Generally, the strength and plasticity of alloys have a contradictory relationship, and it is challenging to increase both the strength and plasticity simultaneously [40]. For the present RHEAs, with the addition of C, besides the improvement of corrosion resistance, the RHEAs showed enhanced hardness, yield strength, and compressive plasticity for all C1 and C3 RHEAs. Especially, as compared with the C0 alloy, the hardness, yield strength, maximum strength, and fracture strain of the C3 alloy were improved to 448 kfg/mm^2^, 1368 MPa, 1904 MPa, and 19.7%, respectively (Table S5). Compared with other WNbMoTa-based and C-doped WNbMoTa RHEAs with a single BCC phase, the developed RHEAs displayed good combinations of strength and plasticity (Table S6). Meanwhile, the RHEAs also possessed high specific yield stress, which indicated good combination of mechanical properties, benefiting their engineering applications, as shown in Figure 6c.

With the dissolution of the C element in both the dendritic and interdendritic regions, the improved strength and hardness of the RHEAs with the C addition may stem from the enhancement of solid solution hardening effect, indicated by the increased *δ* from C addition [43,45]. The solid solution strengthening in RHEAs could be described by the models proposed by Senkov et al. [46], which considers the atomic size mismatch and shear moduli mismatch, as shown by the following equation:

(6) $$\sigma_{s,i}=AG\;c_{i}^{\frac{2}{3}}\delta_{i}^{\frac{4}{3}}$$

where *σ*_s,i_ is the strengthening resulted from the *i*th element; *A* is a parameter related to the materials, which was in order of 0.04; *G* is the shear modulus of the materials; and *c*_i_ is the atomic percentage of the ith element. *δ*_i_ is a parameter considered the atomic size mismatch and shear moduli mismatch, which can be expressed by the equation:

(7) $$\delta_{i}=\;{\xi \left(\delta G_{i}^{2}\;+\;\beta \delta r_{i}^{2}\right)}^{\frac{1}{2}}$$

where *ξ* is equal to 2.5 for BCC materials; *β* is equal to 16 in most HEAs; and *δG*_i_ and *δr*_i_ are the shear moduli mismatch and atomic size mismatch induced from the *i*th element. Based on the Moreen’s hypothesis, an effective model was utilized to express the *δG*_i_ and *δr*_i_, which could be calculated by the following equations [47]:

(8) $${\delta r}^{ave}=\;\overset{n}{\underset{i}{\sum }}\overset{n}{\underset{j}{\sum }}c_{i}c_{j}{\delta r}_{I,j}$$

(9) $${\delta G}^{ave}=\overset{n}{\underset{i}{\sum }}\overset{n}{\underset{j}{\sum }}c_{i}c_{j}{\delta G}_{i,j}$$

**Figure 7:**
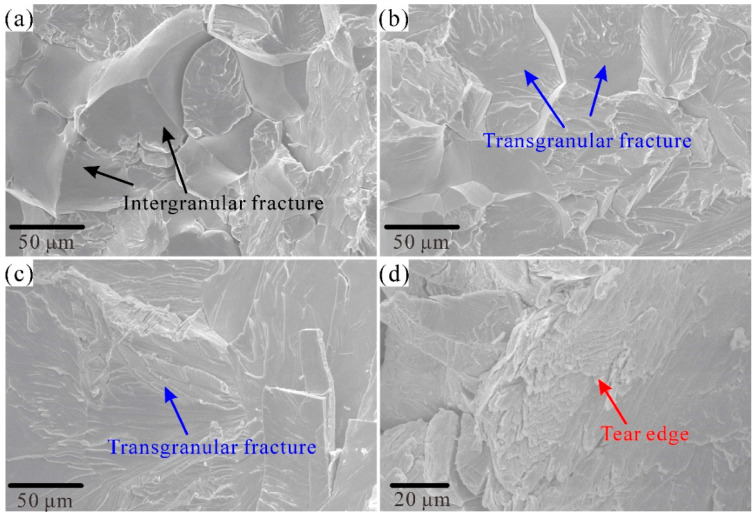
Fracture morphology of the C0 (**a**,**b**) and C3 (**c**,**d**) RHEAs. A mixed fracture model including intergranular fracture (**a**) and transgranular fracture (**b**) was found in the C0 RHEA, while for C3 RHEA, only mainly a transgranular fracture (**c**) was found. (**d**) The tear edge at a high magnification of the C3 alloy, suggesting the occurrence of better plastic deformation.

Therefore, the atomic size mismatch and shear modulus mismatch induced by the interstitial atom (C) in the C-doped RHEAs could be calculated by following equations:

(10) $${\delta r}_{i}=\;\frac{{\delta r}_{\mathrm{CRHEA}}^{ave}{-\delta r}_{\mathrm{RHEA}}^{ave}}{\delta C}$$

(11) $${\delta G}_{i}=\frac{{\delta G}_{\mathrm{CRHEA}}^{ave}{-\delta G}_{\mathrm{RHEA}}^{ave}}{\delta C}$$

where *δ*C is the atomic fraction difference in C between the C-doped RHEA and the C-free RHEA. It was demonstrated that doping C could result in a strong interstitial solid-solution strengthening in the C-doped RHEAs due to the fact that the C element exhibited a much smaller atomic radius (about 77 pm) compared to that of other constituent elements, for example, 146 pm of Ti. According to Equation 6, the strengthening from interstitial solutions was closely related to the differences between the mismatch of the atomic radius. The great differences between the atomic radii between C and Ti further confirms such a strengthening effect according to Equations (10) and (11). Moreover, the strengthening mechanisms of C1 and C3 could vary according to the results of the microstructural analysis. Compared to C0, the precipitation-strengthening mechanism, resulting from the formation of C-rich precipitation (Figure S1), could contribute to the improved strength in the C1 alloy. For RHEAs with both dendrite and interdendrite regions, with different physical properties, the stress tended to concentrate at the soft interdendrite region [42]. For the present RHEAs, due to the concentrations of different elements within the dendrite and interdendrite, the addition of the C element may strengthen the interdendrite region, allowing better plastic deformation. The enrichment of C at the grain boundary has been proven to play a key role in strengthening the grain boundary of WNbMoTa, resulting in the improvement of both strength and plasticity [16]. According to the EDS results shown in Figure S1, it was obvious that a higher concentration of C was found at the grain boundary of C3, which may also indicate a similar strengthening mechanism existed in C3. To reveal the strengthening mechanisms from adding C, the fracture morphology of C0 and C3 was analyzed. As presented in Figure 7a,b, a mixture fracture mode including intergranular and transgranular fractures was found in the C0 alloy. Increasing the C content led the transgranular fracture to be the primary fracture characteristic in C3 (Figure 7c). As the C element played a critical role in improving the grain boundary cohesion [16], the increased strength and plasticity of C3 could be due to the grain boundary strengthening and precipitation from the C addition. Moreover, as shown in Figure 7d, the quasi-cleavage fracture characteristic of tear edge was also detected on the fracture surface of C3. The occurrence of the tear edge suggested more plastic deformation was activated [48], which further proves the improvement in plastic deformation capability resulting from C addition. It should be pointed out that, for the precipitation-strengthening effect, the elucidation of the detailed crystal structures of the C-rich particles may be useful to discover more about the strengthening mechanisms in future.

Combined with excellent corrosion resistance in high-concentration Cl^−^ concentrations with high strength, the development of Nb_25_Mo_25_Ta_25_Ti_20_W_5_C*_x_* RHEAs provides possible solutions to resolve the Cl^−^ attack issue in many potential fields, including marine engineering, power plants, and other corrosive environments. Moreover, since RHEAs also demonstrate superior phase stability, radiation resistance, and oxidation resistance, they may also be used for the next generation of nuclear plants and engines [49]. Understanding the corrosion behavior of RHEAs will also be helpful for the processing of RHEA components using electrochemical techniques [50].

## 4. Conclusions

In summary, single-phase Nb_25_Mo_25_Ta_25_Ti_20_W_5_C*_x_* (*x* = 0.1, 0.3, 0.8 at.%) RHEAs were developed, demonstrating excellent corrosion resistance in high-concentration NaCl solutions. The main findings are as follows:

(1) Differing from the conventional aluminum alloys, titanium alloys, magnesium alloys, as well as stainless steels, the high corrosion resistance of the developed Nb25Mo25Ta25Ti20W5Cx maintained or even improved with the significant increase in electrolyte concentrations, as confirmed by potentiodynamic polarization, EIS, the corroded morphology, and the current–time results. In the 23.5 wt.% NaCl solution, the *i*corr and *E*t reached the lowest and highest value, respectively, for all the C-doped alloys. Typically, the *i*corr (2.36 × 10−7 A/cm2) of the typical Nb25Mo25Ta25Ti20W5C0.1 RHEA is nearly two orders lower than that of 304 L stainless steel (1.75 × 10−5 A/cm2), demonstrating superior corrosion resistance in the high-concentration NaCl solution.
(2) The sustainable high corrosion resistance of the Nb25Mo25Ta25Ti20W5C*x* RHEAs was attributed to the fewer defects as well as the formation of stable oxides in the passive film. For the typical Nb25Mo25Ta25Ti20W5C0.1 RHEA, more elements with high valence states were found in the 13.5 wt.% and 23.5 wt.% NaCl solutions, where more stable oxides in the passive film formed. Additionally, no obvious corrosion pit was found on the surface of this alloy after immersing it in NaCl solutions (13.5 wt% and 23.5wt%) for 2.5 h.
(3) After the addition of the C element, the Nb25Mo25Ta25Ti20W5C*x* RHEAs demonstrated increased strength and plasticity simultaneously, where the Nb25Mo25Ta25Ti20W5C0.3 alloy showed an average yield strength of 1368 MPa and plastic strain of 19.7%. Such phenomena were mainly attributed to the solid solution-strengthening, grain boundary-strengthening, and precipitation-strengthening effects. The present findings provide possible solutions for the development of highly corrosion-resistant alloys to address the Cl− electrochemical corrosion issue in high-strength alloys for harsh environments.

## Data Availability

The data supporting this article have been included as part of the Supplementary Information. The data presented in this study are available on request from the corresponding author.

## Supplementary Materials

The following supporting information can be downloaded at: https://www.mdpi.com/article/10.3390/e28010105/s1. Table S1. Calculated empirical parameters of the Nb_25_Mo_25_Ta_25_Ti_20_W_5_C*_x_* RHEAs. Table S2. Corrosion parameters of 304 L, C0, C1, C3, and C8 alloys obtained from the potentiodynamic polarization curves, including corrosion current density (*i*_corr_), corrosion potential (*E*_corr_), over-passivation potential (*E*_t_), and pitting potential (*E*_pit_). Table S3. EEC parameters for impedance spectra of C0, C1, C3, C8, and 304 L in different NaCl solutions at 35 °C. Table S4. The relative content of W, Nb, Mo, Ta, and Ti under different states in the passive film formed on the C1 RHEA surface in a 0 wt.%, 3.5 wt.%, 13 wt.%, and 23.5 wt.% NaCl solutions (35 °C and immersing time of 2.5 h). Table S5. Mechanical properties of C0, C1, C3, and C8 RHEAs, including yield strength (*σ*_y_), maximum strength (*σ*_max._), plastic strain (*ε*_f_), and Vickers hardness (*H_v_*). Table S6. Comparison of the mechanical properties of single-phase WNbMoTa-based and C-doped WNbMoTa RHEAs, including the yield strength (*σ*_y_), density (*ρ*), specific yield strength (*SYS*), and plastic strain (*ε*_f_). Figure S1. EDS point results at point 1 (a), point 2 (b), and point 3 (c) for the C1, C3, and C8 RHEAs, respectively. Figure S2. Potentiodynamic polarization curves of C0 (a), C3 (b), and C8 (c) RHEAs in the 3.5 wt.%, 13.5 wt.%, and 23.5 wt.% NaCl solutions. Similarly to C1 in Figure S2a, the C0, C3, and C8 alloys also show obviously second passive region and no dramatic current increase. Figure S3. EIS plots of the C0, C3, and C8 RHEAs in the 3.5 wt.%, 13.5 wt.%, and 23.5 wt.%, NaCl solutions, including the Nyquist curves (a–c), Bode magnitude plots (d–f), and Bode phase plots (g–i). Figure S4. Typical morphology of the C3 and C8 RHEAs after potentiodynamic polarization in NaCl solutions. Figure S5. Current–time curves of C0 RHEA in the 3.5 wt.%, 13.5 wt.%, and 23.5 wt.% NaCl solutions.
