# Supplementary material for: Sustainable High Corrosion Resistance in High-Concentration NaCl Solutions for Refractory High-Entropy Alloys with High Strength and Good Plasticity
Source: Entropy (Basel). 2026 Jan 15;28(1):105. doi: 10.3390/e28010105 (PMC12839976; doi:10.3390/e28010105)
Supplement: Supplementary file 1 [file entropy-28-00105-s001.zip › entropy-4063051-supplementary.pdf]

entropy

Supplementary Information for

Sustainable High Corrosion Resistance in High-Concentration NaCl Solutions for Refractory High-Entropy Alloys with High Strength and Good Plasticity

Shunhua Chen [1,2,*], Xinxin Liu [1], Chong Li [1], Wuji Wang [1] and Xiaokang Yue [1,2]

[1] School of Mechanical Engineering, Hefei University of Technology, Hefei 230009, China
[2] Key Laboratory of Advanced Functional Materials and Devices of Anhui Province, Hefei University of Technology, Hefei 230009, China
* Correspondence: shchen@hfut.edu.cn

List of Tables

Table S1. Calculated empirical parameters of the $Nb_{25}Mo_{25}Ta_{25}Ti_{20}W_5C_x$ RHEAs.

Table S2. Corrosion parameters of 304L, C0, C1, C3 and C8 alloys obtained from the potentiodynamic-polarization curves, including corrosion current density (i_{corr}), corrosion potential (E_{corr}), over-passivation potential (E_t), and pitting potential (E_{pit}).

Table S3. EEC parameters for impedance spectra of C0, C1, C3, C8 and 304L in different NaCl solutions at 35 ℃.

Table S4. The relative content of W、Nb、Mo、Ta and Ti under different states in the passive film formed on the C1 RHEA surface in a 0 wt.%, 3.5 wt.%, 13 wt.% and 23.5 wt.% NaCl solutions (35 ℃ and immersing time of 2.5 h).

Table S5. Mechanical properties of C0, C1, C3 and C8 RHEAs, including yield strength (σ_y), maximum strength ($\sigma_{max.}$), plastic strain (ε_f), and Vickers hardness (H_v).

Table S6. Comparison of the mechanical properties of single-phase WNbMoTa-based and C-doped WNbMoTa RHEAs, including the yield strength (σ_y), density (ρ), specific yield strength (SYS) and plastic strain (ε_f).

List of Figures

Figure S1. EDS point results at point 1 (a), point 2 (b) and point 3 (c) for C1, C3 and C8 RHEAs, respectively.

Figure S2. Potentiodynamic polarization curves of C0 (a), C3 (b) and C8 (c) RHEAs in the 3.5 wt.%, 13.5 wt.% and 23.5 wt.% NaCl solutions. Similar to C1 in Figure 2a, C0, C3 and C8 alloys also show obviously second passive region and no dramatic current increase.

Figure S3. EIS plots of C0, C3 and C8 RHEAs in the 3.5 wt.%, 13.5 wt.%, 23.5 wt.% NaCl solutions, including the Nyquist curves (a,b,c), Bode magnitude plots (d,e,f), and Bode phase plots (g,h,i).

Figure S4. Typical morphology of C3 and C8 RHEAs after potentiodynamic polarization in NaCl solutions.

Figure S5. Current-time curves of C0 RHEA in the 3.5 wt.%, 13.5 wt.% and 23.5 wt.% NaCl solutions.

Supplementary S1

Table S1. Calculated empirical parameters of the $Nb_{25}Mo_{25}Ta_{25}Ti_{20}W_5C_x$ RHEAs.

Alloy	ΔS_{mix}[J/(k·mol)]	ΔH_{mix}(kJ/mol)	δ(%)	*VEC*	*Ω*
C0	1.511R	-3.940	2.6	5.100	8.953
C1	1.518R	-4.301	3.0	5.099	8.239
C3	1.527R	-5.021	3.6	5.097	7.107
C8	1.546R	-6.782	4.8	5.091	5.333

Table S2. Corrosion parameters of 304L, C0, C1, C3 and C8 alloys obtained from the potentiodynamic-polarization curves, including corrosion current density (i_{corr}), corrosion potential (E_{corr}), over-passivation potential (E_t), and pitting potential (E_{pit}).

Alloy	Concentration(wt.%)	I_{corr}(A/cm^2)	E_{corr}(V vs. SCE)	E_t(V vs. SCE)	E_{pit}(V vs. SCE)
304L	3.5	4.98E-6±9.09E-7	-0.415±6.92E-4	0.330±4.05E-3	0.375±4.08E-3
	13.5	1.36E-5±2.57E-6	-0.203±1.47E-4	0.025±4.07E-3	-0.050±4.09E-3
	23.5	1.96E-5±3.12E-6	-0.179±2.76E-4	0.040±4.08E-3	-0.100±4.10E-3
C0	3.5	4.13E-6±2.56E-7	-0.608±4.34E-3	1.280±6.02E-3	1.726±5.33E-2
	13.5	8.00E-6±7.08E-7	-0.598±1.88E-3	1.293±1.69E-2	2.039±4.58E-2
	23.5	6.19E-6±8.71E-7	-0.605±4.11E-3	1.368±2.46E-2	2.226±4.54E-2
C1	3.5	9.36E-7±1.52E-7	-0.667±1.25E-3	1.256±8.55E-3	2.303±8.17E-3
	13.5	8.45E-7±1.47E-7	-0.743±9.63E-4	1.314±1.78E-2	2.523±4.09E-3
	23.5	3.88E-7±1.06E-7	-0.744±1.36E-3	1.366±6.22E-3	2.803±4.12E-3
C3	3.5	1.61E-6±2.89E-7	-0.673±2.27E-3	1.107±1.04E-2	1.830±6.24E-3
	13.5	1.00E-6±2.21E-7	-0.724±8.50E-4	1.304±1.22E-2	2.305±6.22E-3
	23.5	7.58E-7±9.32E-8	-0.699±2.85E-4	1.357±1.65E-2	2.608±1.63E-2
C8	3.5	4.82E-6±1.22E-6	-0.654±5.85E-4	1.078±1.25E-2	1.652±6.23E-3
	13.5	6.05E-6±8.74E-7	-0.698±7.39E-4	1.304±3.97E-3	1.855±8.51E-3
	23.5	3.68E-6±9.31E-7	-0.684±1.05E-3	1.371±6.18E-3	1.997±1.02E-2

Table S3. EEC parameters for impedance spectra of C0, C1, C3, C8 and 304L in different NaCl solutions at 35 ℃.

Alloy	C (wt.%)	R_s (Ω·cm²)	R_p (Ω·cm²)	Q1 (μF/cm²)	n_1	R_{ct} (Ω·cm²)	Q2 (μF/cm²)	n_2	Chi-Squared χ^2
304L	3.5	1.92±1.00E-01	1702.00±35	2.66E-05±1.42E-7	0.87±0.001	3.16E+05±1.26E+03	2.97E-05±1.21E-07	0.69±1.00E-03	2.67E-03
	13.5	0.72±3.51E-05	6.61±0.009	1.05E-05±1.3E-08	0.99±0.001	8.15E+02±5.29E-01	9.49E-05±6.00E-09	0.66±9.00E-05	6.60E-03
	23.5	0.57±1.95E-03	5.36±0.006	1.05E-05±8E-07	0.99±0.005	4.24E+02±1.00E-01	1.20E-04±1.90E-06	0.63±7.28E-03	6.68E-03
C0	3.5	1.85±5.03E-03	79.78±4.00	8.09E-05±3.36E-6	0.83±0.003	9.67E+12±1.91E+11	6.79E-04±1.05E-6	0.51±4.79E-04	1.38E-01
	13.5	0.67±2.79E-03	50.42±6.58	6.43E-05±9.02E-07	0.87±0.001	2.50E+06±4.77E+04	1.26E-03±4.62E-07	0.52±4.33E-04	3.20E-02
	23.5	0.44±5.80E-04	37.11±1.54	8.13E-05±3.69E-07	0.83±0.001	8.45E+06±5.13E+02	1.37E-03±5.77E-08	0.52±9.87E-05	9.10E-02
C1	3.5	1.91±5.29E-03	1226.60±52.72	3.48E-05±7.75E-07	0.90±0.003	8.61E+04±7.33E+02	7.74E-05±5.26E-07	0.72±2.52E-03	4.70E-03
	13.5	0.68±3.33E-03	1043.50±99.86	3.61E-05±1.29E-06	0.93±0.004	7.28E+04±1.26E+03	8.55E-05±6.67E-07	0.66±3.62E-03	9.00E-03
	23.5	0.49±1.26E-03	1172.00±64.52	2.05E-05±5.73E-07	0.96±0.002	1.09E+05±1.14E+03	4.27E-05±5.00E-07	0.61±1.33E-04	6.00E-03
C3	3.5	1.90±1.61E-02	431.60±13.69	4.36E-05±1.59E-06	0.87±0.004	1.46E+05±5.11E+03	2.34E-04±1.05E-06	0.72±2.52E-03	3.50E-03
	13.5	0.67±5.87E-03	650.20±62.22	3.62E-05±2.14E-06	0.92±0.007	1.16E+05±8.63E+03	1.61E-04±8.38E-07	0.66±6.31E-03	5.30E-03
	23.5	0.50±8.93E-04	734.00±37.45	2.56E-05±3.28E-07	0.95±0.001	1.70E+05±4.53E+03	1.06E-04±1.46E-07	0.53±1.53E-03	9.40E-03
C8	3.5	1.84±1.73E-03	68.02±0.26	1.21E-04±1.15E-06	0.80±0.001	1.15E+20±8.57E+18	1.14E-03±7.57E-07	0.73±2.95E-04	6.60E-03
	13.5	0.64±1.35E-02	93.85±10.02	6.09E-05±1.09E-05	0.87±0.001	1.83E+20±2.99E+18	1.11E-03±5.83E-06	0.60±6.36E-03	9.40E-03
	23.5	0.46±2.49E-03	133.00±5.38	3.92E-05±1.47E-06	0.91±0.004	1.22E+20±1.10E+19	1.06E-04±1.79E-06	0.52±1.80E-03	6.90E-03

Table S4. The relative content of W、Nb、Mo、Ta and Ti under different states in the passive film formed on the C1 RHEA surface in air and in 3.5 wt.%, 13 wt.% and 23.5 wt.% NaCl solutions (35 ℃ and immersing time of 2.5 h).

Element	Valence state	Relative content (at.%)				Nominal content (at.%)
		In air	3.5 wt.%	13.5 wt.%	23.5 wt.%	
Nb	Nb^0	14.15	9.12	8.01	7.75	25
	Nb^{5+}ox (Nb_2O_5)	4.8	17.97	17.84	18.51	
Mo	Mo^0	15.25	8.63	10.55	8.08	25
	Mo^{4+}ox (MoO_2)	17.69	19.53	12.7	14.91	
	Mo^{6+}ox (MoO_3)	0	0	2.55	2.05	
Ta	Ta^0	17.4	9.41	9	9.61	25
	Ta^{5+}ox (Ta_2O_5)	3.88	14.6	17.96	16.94	
Ti	Ti^0	14.93	3.75	2.89	3.87	20
	Ti^{4+}ox (TiO^2)	2.81	6.32	9.55	8.5	
W	W^0	6.35	4.65	3.06	3.19	5
	W^{4+}ox (WO_2)	2.76	6.02	5.9	6.14	

Table S5. Mechanical properties of C0, C1, C3 and C8 RHEAs, including yield strength (σ_y), maximum strength ($\sigma_{max.}$), plastic strain (ε_f), and Vickers hardness (*Hv*).

	σ_y (MPa)	$\sigma_{max.}$ (MPa)	ε_f (%)	*Hv* (kfg/mm^2)
C0	1308±4	1612±7	13.69±0.43	423±10
C1	1332±25	1751±47	15.41±0.86	438±6
C3	1368±28	1904±72	19.72±1.19	448±13
C8	1365±9	1804±99	15.2±1.71	453±11

Table S6. Comparison of the mechanical properties of single-phase WNbMoTa-based and C-doped WNbMoTa RHEAs, including the yield strength (σ_y), density (ρ), specific yield strength (*SYS*) and plastic strain (ε_f).

RHEA	σ_y(MPa)	ρ(g/cm^3)	*SYS*(MPa·cm^3/g)	ε_f(%)	Ref.
C0	1308±4	10.71	122	13.69±0.43	This work
C1	1332±25	10.71	124	15.41±0.86	This work
C3	1368±28	10.70	128	19.72±1.19	This work
C8	1365±9	10.68	128	15.2±1.71	This work
WNbMoTa	1058	13.62	78	2.1	[1]
WNbMoTaV	1246	12.34	101	1.7	[1]
WNbMoTaTi	1343	11.73	114	14.1	[2]
WNbMoTaVTi	1515	10.94	138	10.6	[2]
WNbMoTa	996	13.62	73	1.9	[3]
$WNbMoTaTi_{0.25}$	1109	13.06	85	2.5	[3]
$WNbMoTaTi_{0.5}$	1211	12.57	96	5.9	[3]
$WNbMoTaTi_{0.75}$	1304	12.13	108	8.4	[3]
WNbMoTaTi	1455	11.73	124	11.5	[3]
$WNbMoTaVZr_{0.1}$	1294	12.17	106	2.0	[4]
$WNbMoTaZr_{0.1}$	1223	13.38	91	6.4	[5]
$(NbMoTaW)_{99.95}C_{0.05}$	1169	13.62	86	6.0	[6]
$(NbMoTaW)_{99.85}C_{0.15}$	1355	13.61	100	8.0	[6]
$(NbMoTaW)_{99.5}C_{0.5}$	1536	13.59	113	5.8	[6]

Supplementary S2

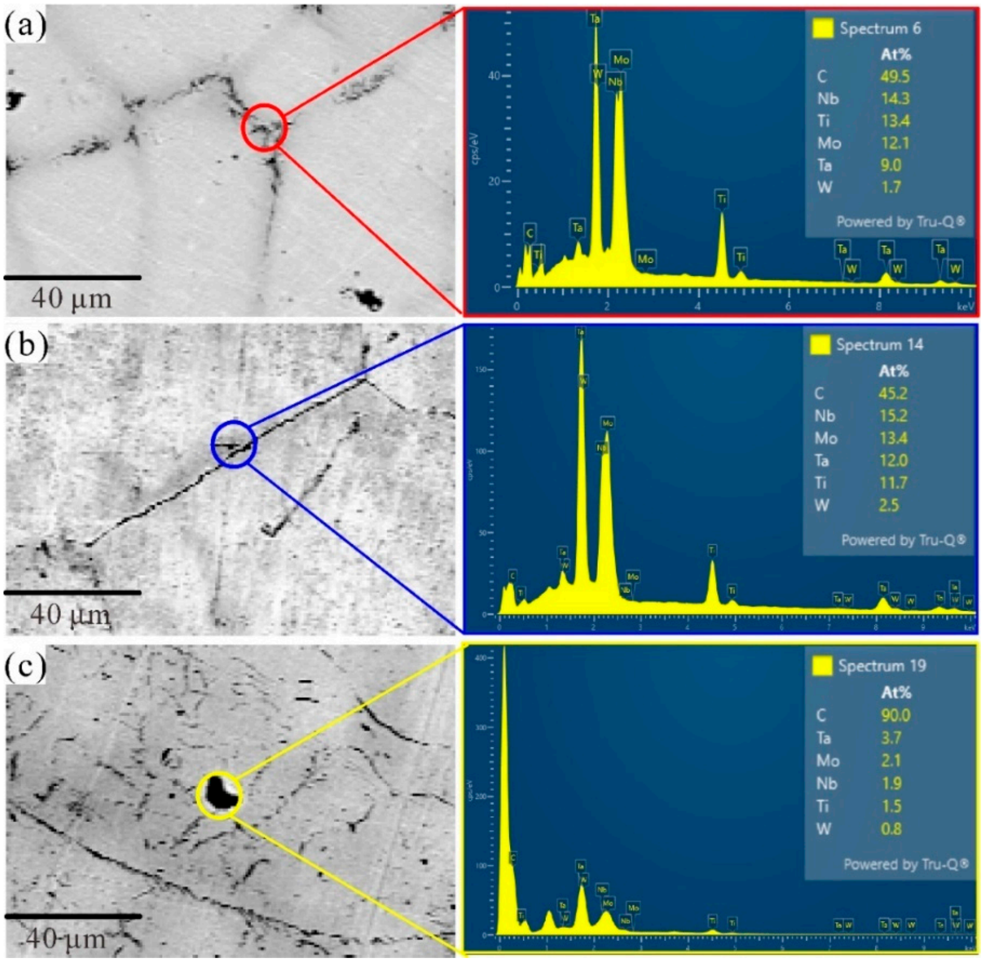

Figure S1. EDS point results at point 1 (a), point 2 (b) and point 3 (c) for C1, C3 and C8 RHEAs, respectively.

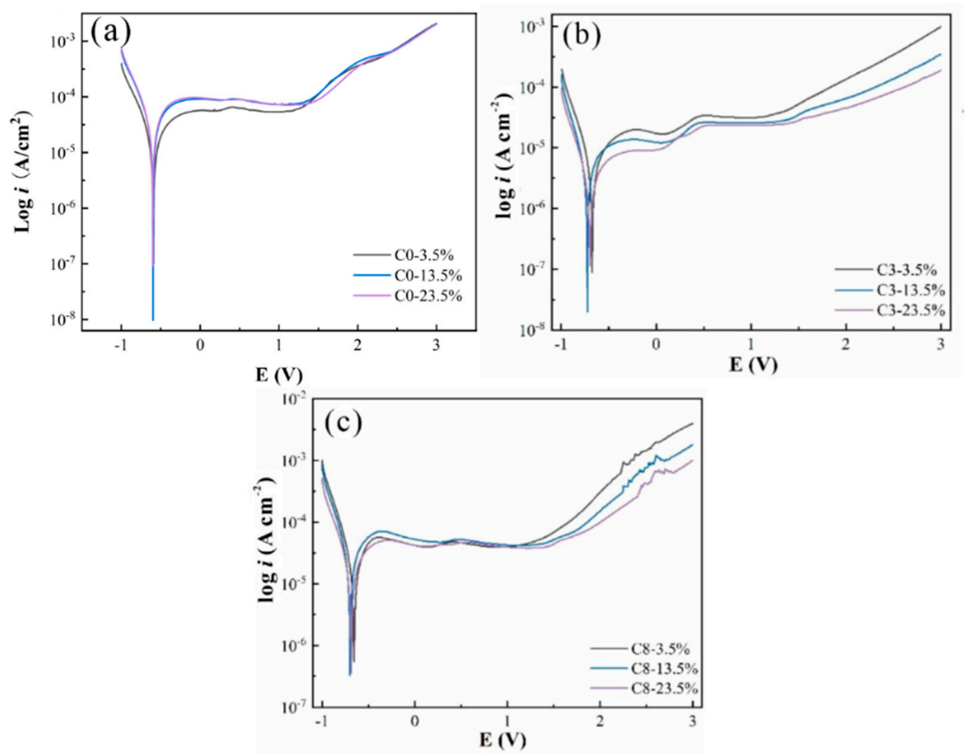

Figure S2. Potentiodynamic polarization curves of C0 (a), C3 (b) and C8 (c) RHEAs in the 3.5 wt.%, 13.5 wt.% and 23.5 wt.% NaCl solutions. Similar to C1 in Figure 2a, C0, C3 and C8 alloys also show obviously second passive region and no dramatic current increase.

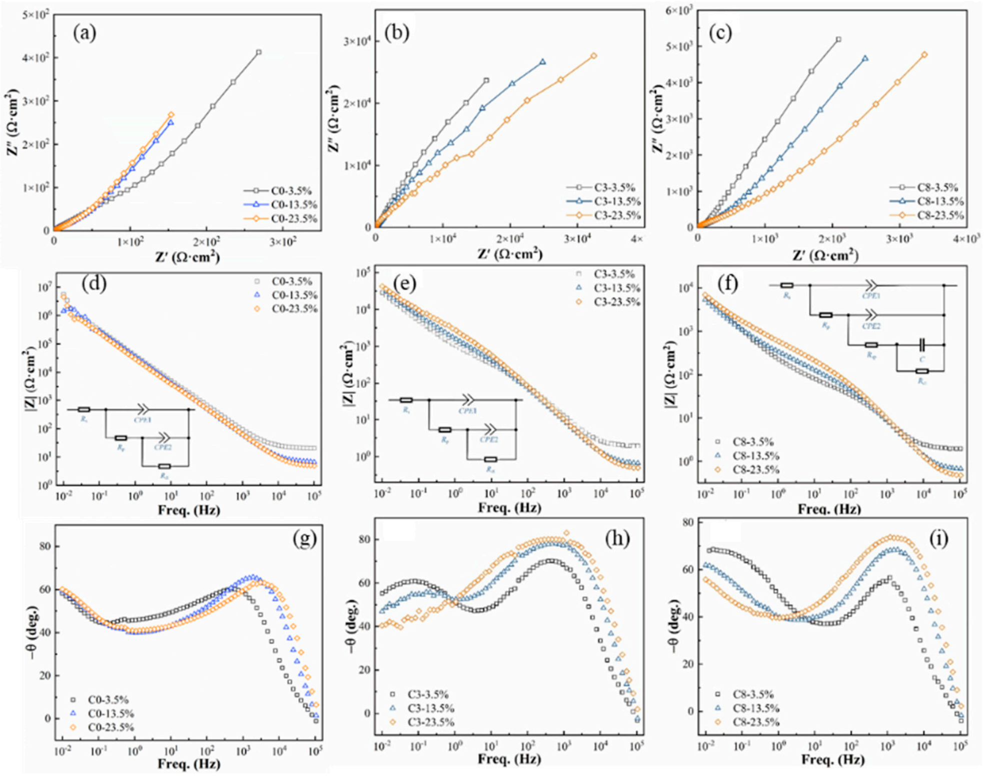

Figure S3. EIS plots of C0, C3 and C8 RHEAs in the 3.5 wt.%, 13.5 wt.%, 23.5 wt.% NaCl solutions, including the Nyquist curves (a,b,c), Bode magnitude plots (d,e,f), and Bode phase plots (g,h,i).

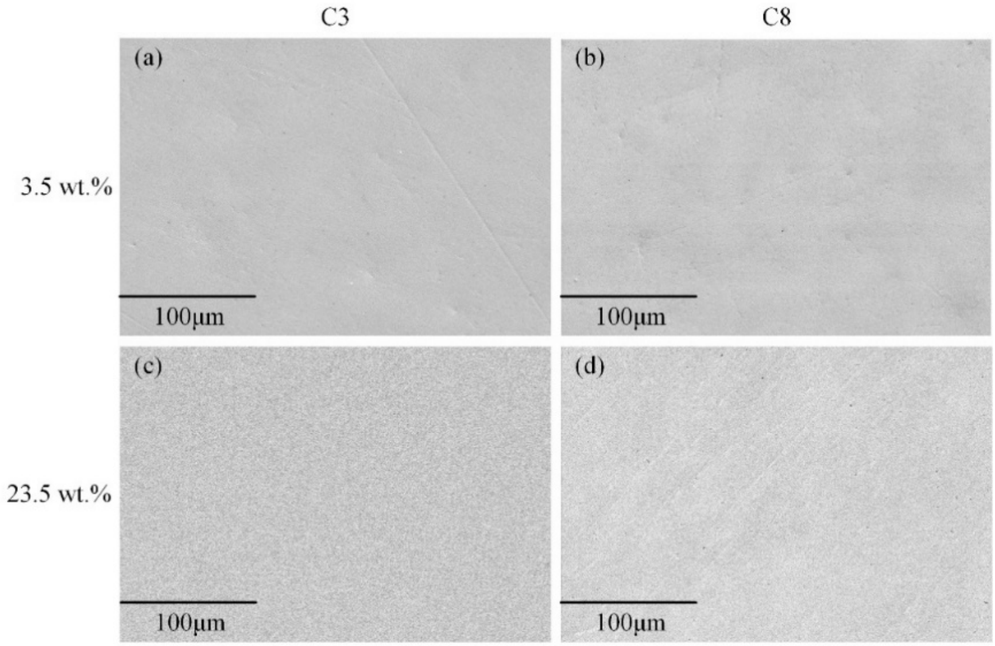

Figure S4. Typical morphology of C3 and C8 RHEAs after potentiodynamic polarization in NaCl solutions.

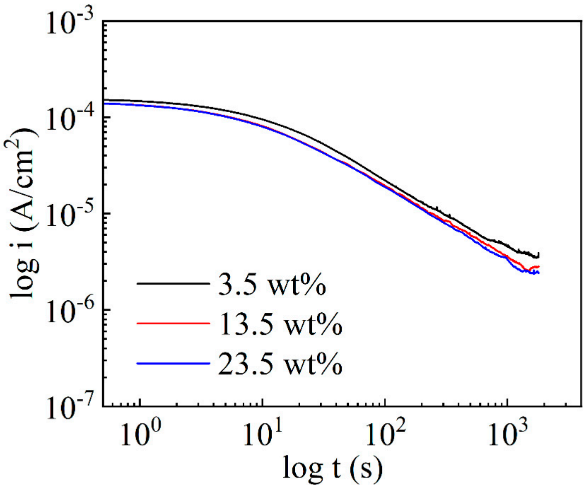

Figure S5. Current-time curves of C0 RHEA in the 3.5 wt.%, 13.5 wt.% and 23.5 wt.% NaCl solutions.

References for Table S6:

1. Senkov, O.N.; Wilks, G.B.; Scott, J.M.; Miracle, D.B. Mechanical Properties of Nb25Mo25Ta25W25 and V20Nb20Mo20Ta20W20 Refractory High-Entropy Alloys. *Intermetallics* **2011**, *19*, 698–706.
2. Han, Z.D.; Chen, N.; Zhao, S.F.; Fan, L.W.; Yang, G.N.; Shao, Y.; Yao, K.F. Effect of Ti Additions on Mechanical Properties of NbMoTaW and VNbMoTaW Refractory High-Entropy Alloys. *Intermetallics* **2017**, *84*, 153–157.
3. Han, Z.D.; Luan, H.W.; Liu, X.; Chen, N.; Li, X.Y.; Shao, Y.; Yao, K.F. Microstructures and Mechanical Properties of Ti NbMoTaW Refractory High-Entropy Alloys. *Materials Science and Engineering A* **2018**, *712*, 380–385.
4. Li, C.; Chen, S.H.; Wu, Z.W.; Zhang, Z.F.; Wu, Y.C. Development of High-Strength WNbMoTaVZrx Refractory High-Entropy Alloys. *Journal of Materials Research* **2022**, *37*, 1664–1678.
5. Chen, S.H.; Zhang, J.S.; Guan, S.; Li, T.; Liu, J.Q.; Wu, F.F.; Wu, Y.C. Microstructure and Mechanical Properties of WNbMoTaZrx (x = 0.1, 0.3, 0.5, 1.0) Refractory High-Entropy Alloys. *Materials Science and Engineering A* **2022**, *835*, 142701.

6. Wang, Z.; Wu, H.; Wu, Y.; Huang, H.; Zhu, X.; Zhang, Y.; Zhu, H.; Yuan, X.; Chen, Q.; Wang, S.; et al. Solving Oxygen Embrittlement of Refractory High-Entropy Alloy via Grain Boundary Engineering. *Materials Today* **2022**, *54*, 83–89.
